# Synthesis and evaluation of the antioxidant activity of 3-pyrroline-2-ones: experimental and theoretical insights†

**DOI:** 10.1039/d2ra04640g

**Published:** 2022-08-30

**Authors:** Nguyen Tran Nguyen, Vo Viet Dai, Adam Mechler, Nguyen Thi Hoa, Quan V. Vo

**Affiliations:** The University of Danang – University of Science and Education Danang 550000 Vietnam ntnguyen@ued.udn.vn; Department of Biochemistry and Chemistry, La Trobe University Victoria 3086 Australia; The University of Danang – University of Technology and Education Danang 550000 Vietnam vvquan@ute.udn.vn

## Abstract

The heterocyclic γ-lactam ring 2-pyrrolidinone has four carbon atoms and one nitrogen atom. Among the group of derivatives of 2-pyrrolidinones, 1,5-dihydro-2*H*-pyrrol-2-ones, also known as 3-pyrroline-2-ones, play a significant structural role in a variety of bioactive natural compounds. In this study, three-component reactions were used to successfully synthesize six polysubstituted 3-hydroxy-3-pyrroline-2-one derivatives. The antioxidant activity of the compounds was tested by the 2,2-diphenyl-1-picrylhydrazyl (DPPH) assay, identifying 4-ethoxycarbonyl-3-hydroxy-5-(4-methylphenyl)-1-phenyl-3-pyrroline-2-one (4b) as the most promising radical scavenger. Quantum chemistry calculations of the thermodynamics and kinetics of the radical scavenging activity also suggest that 4b is an effective HO˙ radical scavenger, with *k*_overall_ values of 2.05 × 10^9^ and 1.54 × 10^10^ M^−1^ s^−1^ in pentyl ethanoate and water, respectively. On the other hand, 4b could not scavenge hydroperoxyl radicals in either media. The ability of 4b to scavenge hydroxyl radicals in polar and non-polar environments is comparable to that of conventional antioxidants such as melatonin, gallic acid, indole-3-carbinol, ramalin, or Trolox. Thus 4b may be classed as a promising HO˙ radical scavenger in the physiological environment.

## Abstract 2

Derivatives of 3-hydroxy-3-pyrroline-2-one were effectively synthesized *via* multicomponent reactions and exhibited potential HO˙ radical scavenging activity.

## Graphical abstract

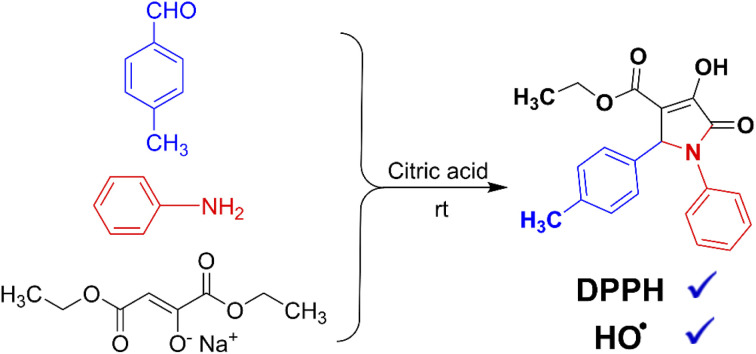

## 1. Introduction

**Fig. 1:**
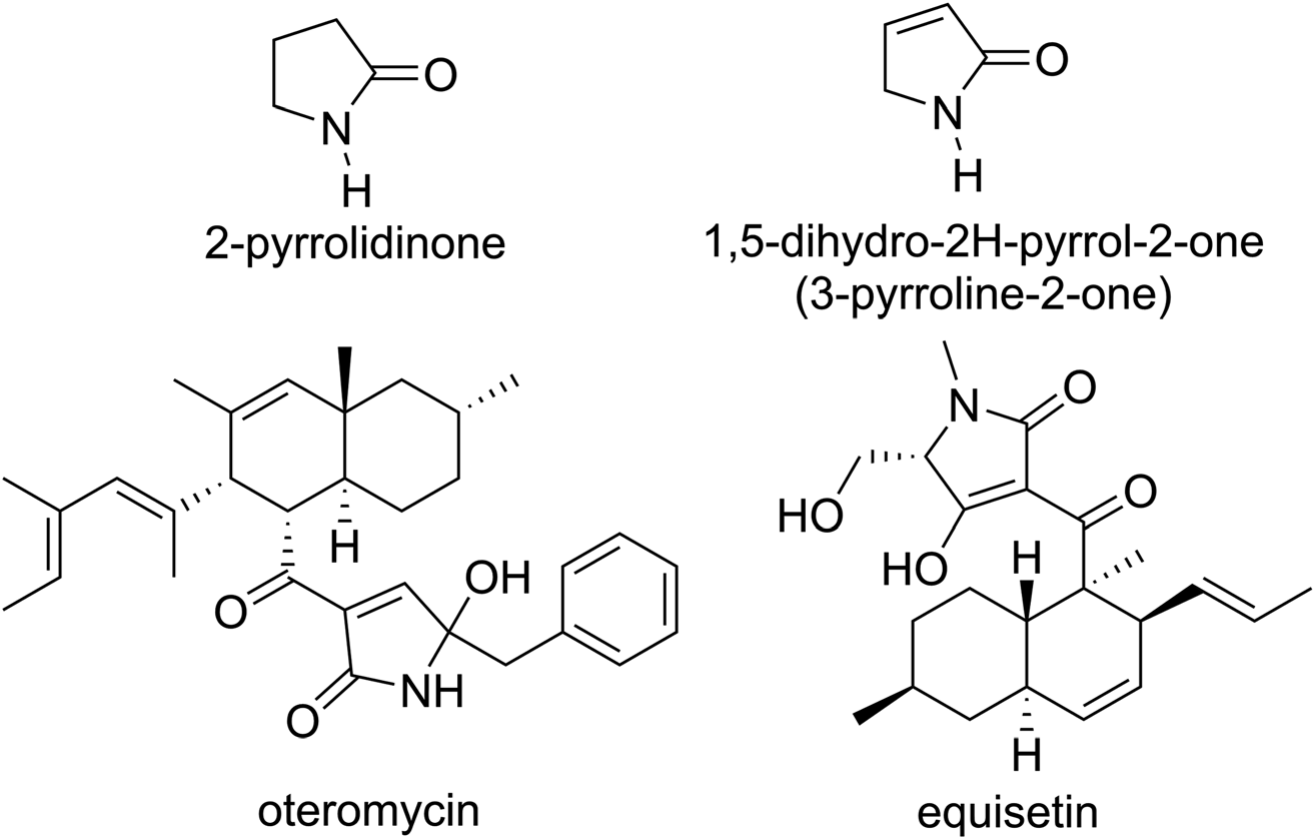
Natural products containing 3-pyrroline-2-one subunit.

2-Pyrrolidinone is a γ-lactam heterocyclic ring containing four carbon atoms and one nitrogen atom. Among the family of 2-pyrrolidinone derivatives, 1,5-dihydro-2*H*-pyrrol-2-ones (also named as 3-pyrroline-2-ones) are important structural subunits of numerous bioactive natural products (Fig. 1). For example, oteromycin that was isolated from fungus strains MF5810 and MF5811 is a HIV-1 integrase inhibitor,^1^ while the *Fusarium pallidoroseum*-originating equisetin is a natural antibiotic active against *Staphylococcus aureus* and *Bacillus subtilis*^2^ while it is also a potent inhibitor against HIV-integrase with IC_50_ values between 7 and 20 µM.^1^ In addition to natural products, more and more non-natural polysubstituted 1,5-dihydro-2*H*-pyrrol-2-ones have also been proven to possess a broad spectrum of pharmacological activities as *e.g.*, anticancer,^3–7^ antibacterial,^8–10^ anti-HIV-1,^11^ as well as anti-inflammatory^12,13^ and antioxidant^2,14–18^ agents. Based on the promising data it was suggested that 1,5-dihydro-2*H*-pyrrol-2-one skeleton-containing heterocyclic compounds are potential drug candidates. Therefore, increasing attention has been directed at the synthesis of 1,5-dihydro-2*H*-pyrrol-2-ones, and especially 3-hydroxy-1,5-dihydro-2*H*-pyrrol-2-ones.^19^

Multi-component reactions (MCRs) offer an efficient synthetic pathway in organic chemistry to obtain complicated molecular structures.^20^ Recently, the synthesis of polysubstituted 3-hydroxy-1,5-dihydro-2*H*-pyrrol-2-ones has been established based on three-component reactions of aromatic aldehydes, arylamines and dialkyl acetylenedicarboxylate.^21–27^ The use of sodium diethyl oxalacetate instead of dialkyl acetylenedicarboxylate in the synthesis of polysubstituted 3-hydroxy-1,5-dihydro-2*H*-pyrrol-2-ones *via* three-component reactions has also been reported; however, there are still some disadvantages regarding to the use of sodium diethyl oxalacetate that need to be improved.^28–30^ Therefore, optimizing reaction conditions to synthesize polysubstituted 3-hydroxy-3-pyrroline-2-ones *via* MCRs involving sodium diethyl oxalacetate is still in demand to the synthetic organic chemist community.

A key bioactivity described above is the potential antioxidant activity of this family of compounds.^2,14–18,31^ The imbalance between the production and consumption of oxidants in biological systems leads to oxidative stress, that is, chemical breakdown/damage of various biologically important molecules.^32^ Although there are natural oxidants of various chemical characteristics, free radicals are the main cause of oxidative stress. They are highly reactive and capable of initiating chain reactions, hence propagating molecular damage. A representative reactive oxygen species is the hydroxyl radical.^33^ It is so reactive that it would effectively assault nearly every organic molecule around the place of its production. This radical is held accountable for the majority of ionizing radiation-induced tissue damage^34^ and is the most significant cause of oxidative DNA damage.^35–37^ Inhibiting OH˙ generation would therefore be an effective means of reducing oxidative stress. However, studies of antioxidant activity of 3-hydroxy-3-pyrroline-2-ones are thus far limited: there is no information on the mechanism and kinetics of the antiradical activity, particularly in physiological environments.

The use of computational approaches to investigate the structure–activity relationship and to direct the design of novel medications with enhanced activity is well established,^38–42^ making it possible to perform the evaluation of the radical (*i.e.*, HO˙ and HOO˙) scavenging activity *in silico*, benchmarked against experimental data.

Thus, in this study, three-component reactions of aromatic aldehydes, amines and sodium diethyl oxalacetate will be optimized in order to shorten reaction time, increase the yields and eliminate the need for elevated temperature. Consecutively, the 1,1-diphenyl-1-picrylhydrazyl (DPPH) free radical scavenging capacity of six polysubstituted 3-hydroxy-3-pyrroline-2-ones will be measured. Finally, the interaction of the best performing 3-hydroxy-3-pyrroline-2-one with HO˙ and HOO˙ radicals will be evaluated using well-established model chemistry based on the quantum mechanics-based test for the overall free radical scavenging activity (QM-ORSA) protocol.^40,43^

## 2. Experimental and computational methods

### 2.1. Experimental

#### 2.1.1. Chemicals and experimental methods

All chemicals were purchased from the main chemical suppliers: Merck, Acros, or Sigma Aldrich. For column chromatography, 70–230 mesh silica 60 (E. M. Merck) was used as the stationary phase. Melting points (not corrected) were determined using a Büchi Melting Point B-545 apparatus. NMR spectra were acquired on Bruker Avance II+ 500 MHz or Bruker Avance II+ 600 MHz instruments and chemical shifts (*δ*) are reported in parts per million (ppm) referenced to tetramethylsilane (TMS) or the internal (NMR) solvent signals. Exact mass measurements were acquired on a quadrupole orthogonal acceleration time-of-flight mass spectrometer (Synapt G2 HDMS, Waters, Milford, MA). Samples were infused at 3 µL min^−1^ and spectra were obtained in positive (or negative) ionization mode with a resolution of 15 000 (FWHM) using leucine enkephalin as lock mass. High-resolution mass measurements were recorded on a SCIEX X500 QTOF with an electrospray ionization source in a positive ion mode. The temperatures of the source were set at 300 °C. Curtain gas chambers were filled with high-purity nitrogen (25 psi). The capillary voltage was constantly kept at 5500 V. The collision energy was set at 10 V and zero collision energy spread. IDA mode was used to find mass in the range (100 to 1000).

#### 2.1.2. General procedure for the synthesis of 1,5-disubstituted-4-ethoxycarbonyl-3-hydroxy-3-pyrroline-2-one

Aromatic aldehyde (1 equiv.), amine (1 equiv.), citric acid (2 equiv.) and absolute ethanol (1.0 mL) were mixed in a round bottom flask. The mixture was magnetically stirred at room temperature under an argon atmosphere for 1 hour. Subsequently, sodium diethyl oxalacetate (2 equiv.) was added and the mixture was stirred vigorously at room temperature under Ar atmosphere for 8 hours and the formation of 1,5-disubstituted-4-ethoxycarbonyl-3-hydroxy-3-pyrroline-2-one was followed by TLC (hexane/EtOAc = 5 : 1 and hexane/EtOAc = 5 : 3.5). Then, CH_2_Cl_2_ and HCl (5%) were added and the resulting mixture was stirred vigorously for 15 minutes. The organic layer was separated, then washed three times with distilled water and dried over MgSO_4_. The crude product was purified *via* recrystallization in the solvent mixture of CH_2_Cl_2_ and absolute ethanol or CH_2_Cl_2_ and ethylacetate to obtain a pure product.

#### 2.1.3. Spectra data of products

##### 2.1.3.1. 1,5-Diphenyl-4-ethoxycarbonyl-3-hydroxy-3-pyrroline-2-one (4a)

M.p. 171–173 °C. ^1^H NMR (500 MHz, CDCl_3_) *δ* 9.14 (s_br_, 1H; –OH), 7.49 (d, ^3^*J*(H,H) = 7.80 Hz, 2H; Ar–H), 7.29–7.23 (m, 7H; Ar–H), 7.10 (t, ^3^*J*(H,H) = 7.47 Hz, 1H; Ar–H), 5.75 (s, 1H), 4.20 (m, 2H; OCH_2_), 1.18 ppm (t, ^3^*J*(H,H) = 7.13 Hz, 3H; CH_3_). ^13^C NMR (125 MHz, CDCl_3_) *δ* 165.21, 162.99, 156.55, 136.36, 135.18, 129.05, 128.68, 128.61, 127.63, 125.93, 122.38, 113.29, 61.68, 61.35, 14.03 ppm. HRMS (ESI-TOF MS/MS) *m*/*z* [M + H]^+^ calcd for C_19_H_17_NO_4_: 324.1236; found: 324.1223.

##### 2.1.3.2. 4-Ethoxycarbonyl-3-hydroxy-5-(4-methylphenyl)-1-phenyl-3-pyrroline-2-one (4b)

M.p. 206–207 °C. ^1^H NMR (500 MHz, CDCl_3_) *δ* 9.09 (s_br_, 1H; –OH), 7.49 (dd, ^3^*J*(H,H) = 8.64 Hz, ^4^*J*(H,H) = 1.08 Hz, 2H; Ar–H), 7.27 (m, 2H; Ar–H), 7.11 (m, 3H; Ar–H), 7.05 (d, ^3^*J*(H,H) = 7.86 Hz, 2H; Ar–H), 5.72 (s, 1H), 4.19 (m, 2H; –OCH_2_–), 2.26 (s, 3H; CH_3_), 1.20 ppm (t, ^3^*J*(H,H) = 7.14 Hz, 3H; CH_3_). ^13^C NMR (125 MHz, CDCl_3_) *δ* 165.21, 193.01, 156.37, 138.36, 136.45, 132.04, 129.39, 129.03, 127.48, 125.85, 122.37, 113.37, 61.45, 61.34, 21.24, 14.07 ppm. HRMS (ESI-TOF MS/MS) *m*/*z* [M + H]^+^ calcd for C_20_H_19_NO_4_: 338.1392; found: 338.1383.

##### 2.1.3.3. 4-Ethoxycarbonyl-3-hydroxy-5-(4-nitrophenyl)-1-phenyl-3-pyrroline-2-one (4c)

M.p. 173–175 °C. ^1^H NMR (500 MHz, CDCl_3_) *δ* 9.05 (s_br_, 1H; –OH), 8.12 (d, ^3^*J*(H,H) = 8.40 Hz, 2H; Ar–H), 7.43 (m, 4H; Ar–H), 7.28 (t, ^3^*J*(H,H) = 7.71 Hz, 2H; Ar–H), 7.13 (t, ^3^*J*(H,H) = 7.43 Hz, 1H; Ar–H), 5.85 (s, 1H), 4.21 (m, 2H; OCH_2_), 1.21 ppm (t, ^3^*J*(H,H) = 7.15 Hz, 3H; CH_3_). ^13^C NMR (125 MHz, CDCl_3_) *δ* 164.66, 162.65, 156.98, 148.16, 142.94, 135.84, 129.44, 128.69, 126.53, 125.07, 122.27, 112.33, 61.78, 60.79, 14.17 ppm. HRMS (ESI-TOF MS/MS) *m*/*z* [M + H]^+^ calcd for C_19_H_16_N_2_O_6_: 369.1087; found: 369.1081.

##### 2.1.3.4. 1-Benzyl-4-ethoxycarbonyl-3-hydroxy-5-(4-methylphenyl)-3-pyrroline-2-one (4d)

M.p. 184–186 °C. ^1^H NMR (500 MHz, CDCl_3_) *δ* 9.05 (s_br_, 1H; –OH), 7.30 (m, 3H; Ar–H), 7.16 (d, ^3^*J*(H,H) = 7.80 Hz, 2H; Ar–H), 7.12 (dd, ^3^*J*(H,H) = 7.94 Hz, ^4^*J*(H,H) = 2.02 Hz, 2H; Ar–H), 6.98 (d, ^3^*J*(H,H) = 8.09 Hz, 2H; Ar–H), 5.18 (d, ^2^*J*(H,H) = 14.79 Hz, 1H; –CH_2_N), 4.84 (s, 1H), 4.08 (m, 2H; OCH_2_), 3.54 (d, ^2^*J*(H,H) = 14.83 Hz, 1H; –CH_2_N), 2.36 (s, 3H; CH_3_), 1.08 ppm (t, ^3^*J*(H,H) = 7.16 Hz, 3H; CH_3_). ^13^C NMR (125 MHz, CDCl_3_) *δ* 165.58, 163.59, 157.75, 138.79, 136.55, 131.53, 129.67, 128.94, 128.69, 127.97, 127.86, 113.43, 61.13, 59.54, 44.01, 21.37, 14.02 ppm. HRMS (ESI-quadrupole) *m*/*z* [M + H]^+^ calcd for C_21_H_21_NO_4_: 352.1549; found: 352.1535.

##### 2.1.3.5. 1-Benzyl-4-ethoxycarbonyl-3-hydroxy-5-(4-nitrophenyl)-3-pyrroline-2-one (4e)

M.p. 224–227 °C. ^1^H NMR (500 MHz, DMSO-d6) *δ* 12.01 (s_br_, 1H; –OH), 8.14 (d, ^3^*J*(H,H) = 8.65 Hz, 2H; Ar–H), 7.40 (d, ^3^*J*(H,H) = 8.57 Hz, 2H; Ar–H), 7.25 (m, 3H; Ar–H), 7.05 (dd, ^3^*J*(H,H) = 7.72 Hz, ^4^*J*(H,H) = 1.88 Hz, 2H; Ar–H), 5.16 (s, 1H), 4.78 (d, ^2^*J*(H,H) = 15.35 Hz, 1H; –CH_2_N), 3.98 (dq, ^3^*J*(H,H) = 7.03 Hz, ^2^*J*(H,H) = 14.09 Hz, 1H; –OCH_2_–), 3.92 (dq, ^3^*J*(H,H) = 6.96 Hz, ^2^*J*(H,H) = 14.00 Hz, 1H; –OCH_2_–), 3.80 (d, ^2^*J*(H,H) = 15.37 Hz, 1H; –CH_2_N), 1.01 ppm (t, ^3^*J*(H,H) = 7.10 Hz, 3H; CH_3_). ^13^C NMR (125 MHz, CDCl_3_) *δ* 164.88, 161.69, 154.09, 147.32, 144.04, 136.20, 129.29, 128.54, 127.77, 127.41, 123.61, 110.97, 59.64, 59.57, 44.18, 13.92 ppm. HRMS (ESI-quadrupole) *m*/*z* [M + H]^+^ calcd for C_20_H_18_N_2_O_6_: 383.1243; found: 383.1231.

##### 2.1.3.6. 4-Ethoxycarbonyl-3-hydroxy-5-(4-methylphenyl)-1-(3-nitrophenyl)-3-pyrroline-2-one (4f)

M.p. 179–181 °C. ^1^H NMR (500 MHz, CDCl_3_) *δ* 9.08 (s_br_, 1H; –OH), 8.30 (t, ^4^*J*(H,H) = 2.17 Hz, 1H; Ar–H), 8.09 (dd, ^3^*J*(H,H) = 8.35 Hz, ^4^*J*(H,H) = 2.29 Hz, 1H; Ar–H), 7.93 (dd, ^3^*J*(H,H) = 8.25 Hz, ^4^*J*(H,H) = 2.14 Hz, 1H; Ar–H), 7.45 (t, ^3^*J*(H,H) = 8.22 Hz, 1H; Ar–H), 7.13 (d, ^3^*J*(H,H) = 8.18 Hz, 2H; Ar–H), 7.08 (d, ^3^*J*(H,H) = 7.93 Hz, 2H; Ar–H), 5.76 (s, 1H), 4.20 (m, 2H; –OCH_2_), 2.67 (s, 3H; CH_3_), 1.20 ppm (t, ^3^*J*(H,H) = 7.11 Hz, 3H; CH_3_). ^13^C NMR (125 MHz, CDCl_3_) *δ* 165.24, 163.12, 156.11, 148.55, 139.05, 137.75, 131.16, 130.00, 129.81, 127.43, 127.34, 120.08, 116.06, 114.01, 61.70, 61.21, 21.29, 14.07 ppm. HRMS (ESI-quadrupole) *m*/*z* [M + H]^+^ calcd for C_20_H_18_N_2_O_6_: 383.1243; found: 383.1232.

#### 2.1.4. DPPH assay

Radical-scavenging properties of polysubstituted 3-hydroxy-3-pyrroline-2-ones were evaluated against 1,1-diphenyl-2-picrylhydrazyl (DPPH) radical.^44–48^ DPPH solution (1 mM) was prepared in methanol and solutions of each polysubstituted 3-hydroxy-3-pyrroline-2-one were prepared in DMSO at various concentrations (128, 32 and 8 µg mL^−1^). Then, 200 µL DPPH solution was added to 1.28 µL samples at each tested concentration and the free radical scavenging reactions were carried out on a 96-well plate at 37 °C for 30 minutes. The absorbance was measured at 517 nm wavelength by a BioTek Epoch 2 Microplate Spectrophotometer. The percentage of free radical scavenging was calculated as SP(%) = [(OD_0_ − OD_1_)/OD_0_] × 100, where OD_0_ was defined as the final absorbance of the control reaction with quercetin as the reference antioxidant, and OD_1_ stands for the absorbance in the presence of the sample. Each experiment was repeated three times and quercetin was used as the positive control.

### 2.2. Computational details

All DFT calculations were carried out with the Gaussian 09 suite of programs.^49^ M06-2X functional^50^ and 6-311++G(d,p) basis set were used for all calculations. The M06-2X functional offers one of the most reliable methods to study the thermodynamics and kinetics of radical reactions.^40,50–55^ The kinetic calculations were performed following the QM-ORSA protocol,^40,43^ following the literature.^54–61^ This method has been repeatedly benchmarked against experimental data, delivering results with low errors (*k*_calc_/*k*_exp_ ratio = 1–2.9), particularly in lipid and aqueous solutions.^40,43,52,62^ As a reference all details of the calculations are shown in Table S1, ESI.†

## 3. Results and discussion

### 3.1. Synthesis of polysubstituted 3-pyrroline-2-ones

The reaction between benzaldehyde (1a), aniline (2a) and sodium diethyl oxalacetate (3) in the presence of citric acid (2 equiv.) as the catalyst was used as the starting point to optimize the reaction conditions. Equimolar amounts of 1a, 2a and 3 at 0.5 M concentrations in absolute ethanol at room temperature turned over to 1,5-diphenyl-4-ethoxycarbonyl-3-hydroxy-3-pyrroline-2-one (4a) with 42% yield. This result is consistent with prior reports.^29,30^

There was a slight decrease in the yield of 4a (35%) when the concentration of starting materials in solvent was reduced to 0.34 M. The yield was only 37–38% when the concentration of 1a or 2a was increased to 0.75 M in absolute ethanol. However, there was a dramatic increase in the yield of 4a to 86% when 2 equiv of sodium diethyl oxalacetate (3) was used (Table 1). Therefore, the ratio 1 : 1 : 2 of reactants aromatic aldehyde, amine and sodium diethyl oxalacetate, respectively, was used to synthesize other polysubstituted 3-hydroxy-1,5-dihydro-2*H*-pyrrol-2-ones in absolute ethanol (1 mL) (Table 2).

**Table 1:** Starting materials 1a : 2a : 3 optimization for the synthesis of 4a

Entry	Solvent volume (mL)	Ratio 1a : 2a : 3 (equiv.)	Concentration 1a : 2a : 3 (mmol mL^−1^)	Yield (%)
1	1.0	1 : 1 : 1	0.5 : 0.5 : 0.5	42
2	1.5	1 : 1 : 1	0.34 : 0.34 : 0.34	35
3	1.0	1.5 : 1 : 1	0.75 : 0.5 : 0.5	37
4	1.0	1 : 1.5 : 1	0.5 : 0.75 : 0.5	38
5	1.0	1 : 1 : 1.5	0.5 : 0.5 : 0.75	68
6	1.0	1 : 1 : 2	0.5 : 0.5 : 1.0	86

**Table 2:** Synthesis of polysubstituted 3-hydroxy-1,5-dihydro-2*H*-pyrrol-2-ones

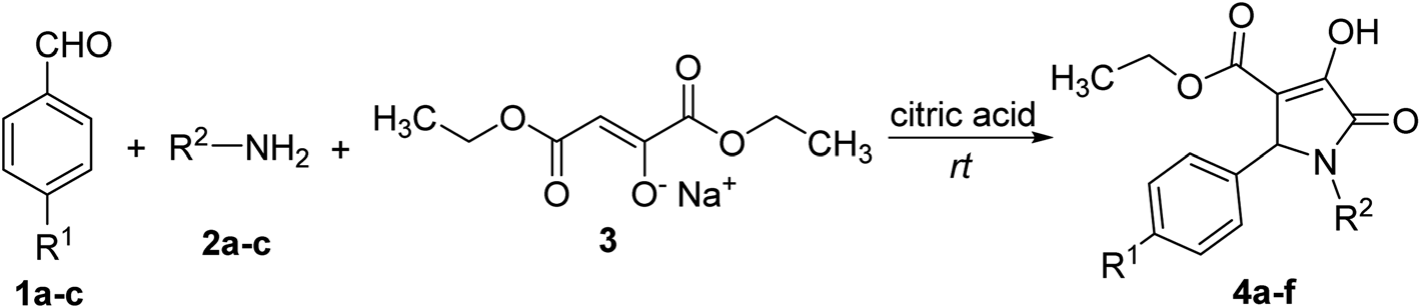				
Entry	R^1^	R^2^	Product	Yield (%)
1	H	C_6_H_5_	4a	86
2	CH_3_	C_6_H_5_	4b	72
3	NO_2_	C_6_H_5_	4c	91
4	CH_3_	C_6_H_5_CH_2_	4d	55
5	NO_2_	C_6_H_5_CH_2_	4e	79
6	CH_3_	3-NO_2_C_6_H_4_	4f	70

**Scheme 1:**
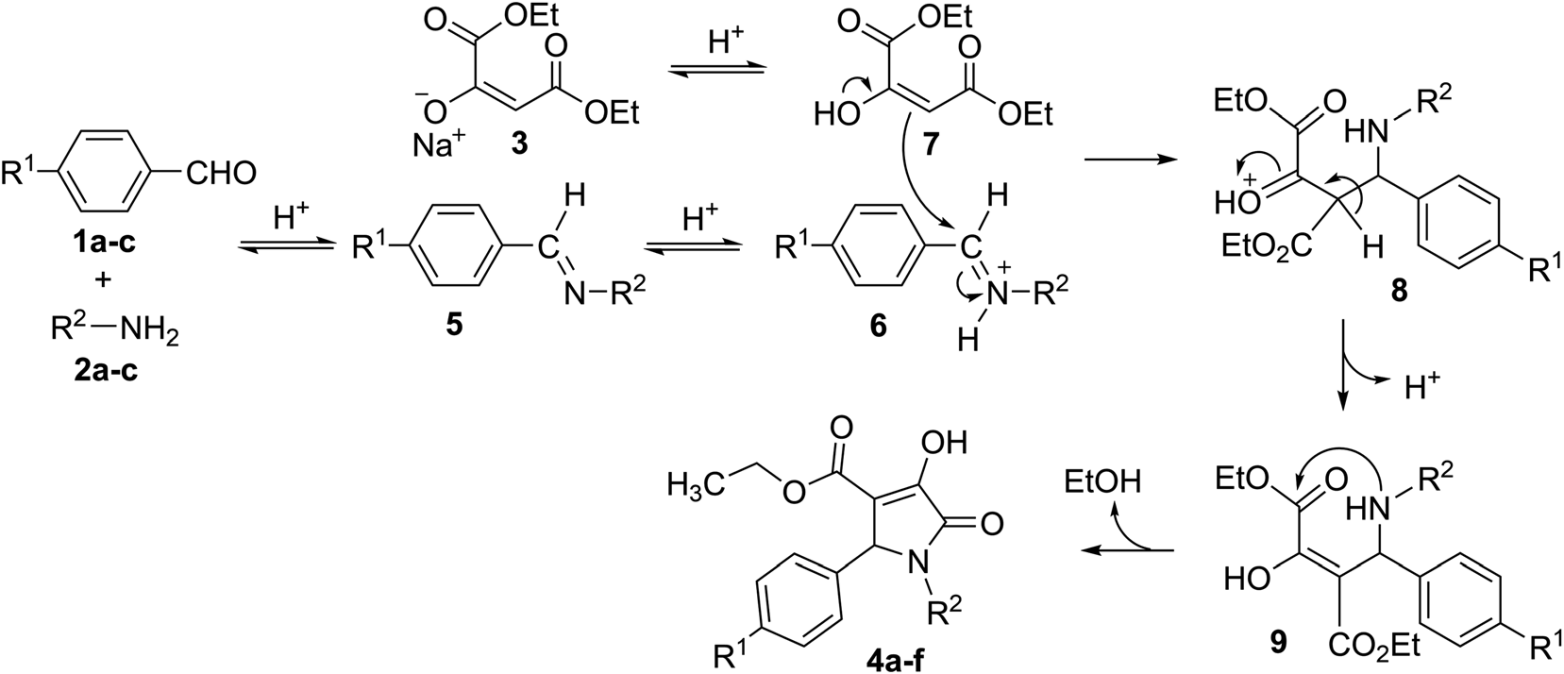
Mechanism for the synthesis of polysubstituted 3-hydroxy-1,5-dihydro-2*H*-pyrrol-2-ones.^19^

The initial step in the multi-component reaction to synthesize polysubstituted 3-hydroxy-1,5-dihydro-2*H*-pyrrol-2-ones proceeds *via* acid-catalyzed condensation of aromatic aldehyde (1a–c) and amine (2a–c) in the presence of citric acid as the catalyst to yield imine (5) which will be then protonated to iminium (6).^21,24^ In the next step, sodium diethyl oxalacetate (3) affords the enol derivative (7) in an acidic environment, and subsequently, enol (7) will react with iminium species 6 to obtain intermediate 8 containing protonated ketone functional group. Then, the deprotonation of 8 will result in intermediate 9 containing enol moiety. The intramolecular nucleophilic attack of the secondary amino group present in intermediate 9 to the carbonyl carbon of a carboxylate moiety results in the formation of polysubstituted 3-hydroxy-1,5-dihydro-2*H*-pyrrol-2-ones (4a–f) (Scheme 1).^19,21,24^

The presence of methyl group in 4-methylbenzaldehyde (1b) will reduce the electrophilicity of carbonyl carbon *via* a positive inductive effect as compared to benzaldehyde. In contrast, the electron-withdrawing property of the nitro group in 4-nitrobenzaldehyde (1c) results in an increase in the electrophilicity of the carbonyl carbon.^63^ Consequently, the rate of imine formation reaction between 4-nitrobenzaldehyde (1c) and aniline (2a) is faster than that between 4-methylbenzaldehyde (1b) and aniline (2a).^64^ The easier the imine formation, the higher the yield of desired product polysubstituted 3-hydroxy-1,5-dihydro-2*H*-pyrrol-2-one. Moreover, the formation of the –CH=N– double bond will decrease when using aliphatic amines as compared to aromatic amines.^65,66^ Therefore, the yields of polysubstituted 3-hydroxy-1,5-dihydro-2*H*-pyrrol-2-ones 4b, 4c are higher than that of 4d, 4e, respectively.

In the structure of polysubstituted 3-hydroxy-1,5-dihydro-2*H*-pyrrol-2-ones, the –C(O)OCH_2_CH_3_ group is almost coplanar with the plane of the 3-pyrroline-2-one.^21,22^ As a consequence, two protons of methylene group are diastereotopic and they will show geminal coupling in ^1^H NMR. In addition, methylene protons (CH_2_) will also couple with protons of methyl group (CH_3_) separated by three sigma bonds. Therefore, two protons of methylene group should be represented by two doublet of quartet (dq) instead of a simple quartet. For instance, ^1^H NMR of 1-benzyl-4-ethoxycarbonyl-3-hydroxy-5-(4-nitrophenyl)-3-pyrroline-2-one (4e) showed two doublet of quartet at the chemical shift of 3.92 and 3.98 ppm representing for two methylene protons of –C(O)OCH_2_CH_3_ group. In addition, two protons of methylene group attached to nitrogen at 1-position are also diastereotopic and thus, represented by two doublets at the chemical shifts of 4.78 and 3.80 ppm.

### 3.2. Radical scavenging activity of 3-pyrroline-2-ones

#### 3.2.1. DPPH antioxidant assay

In the initial evaluation of the antioxidant activity of the synthetic 3-pyrroline-2-ones, the DPPH assay was used following the literature^44,47,48^ using quercetin as the reference antioxidant (Table 3). Under the tested conditions, the studied compound exhibited lower DPPH scavenging activity (EC_50_ > 128 µg mL^−1^, 0.33–0.39 mM) than quercetin (9.97 ± 0.25 µg mL^−1^, 0.033 mM). Among the synthetic compounds, the best DPPH antiradical activity was observed for 4b (40% DPPH at 128 µg mL^−1^), which was more than five times higher than that of 4d (7% DPPH) or 4f (6% DPPH). Activities of 4a, 4c and 4e were intermediate at 14, 32 and 26% DPPH, respectively. However, previous studies showed that the 3-pyrroline-2-one derivatives could exhibit potent radical scavenging activity in more biologically relevant settings (*i.e.*, against HO˙ or lipid peroxidation)^2,15–18^ thus the HO˙ and HOO˙ radical scavenging activity of the most active compound (*i.e.*, 4b) in physiological environments has been investigated further using computational chemistry.

**Table 3:** DPPH radical scavenging activity

Compound	DPPH (%)			EC_50_ (µg mL^−1^)
	128 (µg mL^−1^)	32 (µg mL^−1^)	8 (µg mL^−1^)	
4a	14	0	0	>128
4b	40	12	0	>128
4c	32	11	0	>128
4d	7	0	0	>128
4e	26	5	0	>128
4f	6	1	0	>128
Quercetin	100	45.5	0	9.97 ± 0.25

#### 3.2.2. Calculations of the HOO˙ and HO˙ radical scavenging activity of the most active compound (4b)

##### 3.2.2.1 The antiradical activity in the gas phase

The antioxidant properties of 4b were initially tested in the gas phase in order to identify the predominant antioxidant mechanism(s) for the more complex calculations in physiological environments. As previously demonstrated, this approach decreases computation time while giving accurate and reliable data.^55^ In the first step, the principal thermodynamic properties (proton affinity (PA), ionization energy (IE) and bond dissociation enthalpy (BDE)) that define the radical scavenging activity mechanisms (sequential proton loss electron transfer (SPLET), single electron transfer proton transfer (SETPT) and formal hydrogen transfer (FHT)),^67,68^ of 4b were computed (Table 4).

**Table 4:** The calculated BDEs, PAs and IEs (in kcal mol^−1^)^a^

Positions	BDE	PA	IE
O3–H	93.2	326.6	186.4
C5–H	76.0		
O7–H	96.1		
C8–H	101.5		
O21–H	89.7		

a 4b numbering is shown in Fig. 3.

The lowest calculated BDE was observed at the C5–H bond at 76.0 kcal mol^−1^, whereas those of the other C(O)–H bonds were higher by about 13.7–25.5 kcal mol^−1^. Thus, data implies that the C5–H bond dominates the antioxidant activity of 4b following the FHT pathway. On the other hand, the radical scavenging activity of 4b according to either the SPLET or SETPT would be challenging due to the high PA and IE values (PA = 326.6 kcal mol^−1^ and IE = 186.4 kcal mol^−1^) in comparison to the BDEs.

**Fig. 2:**
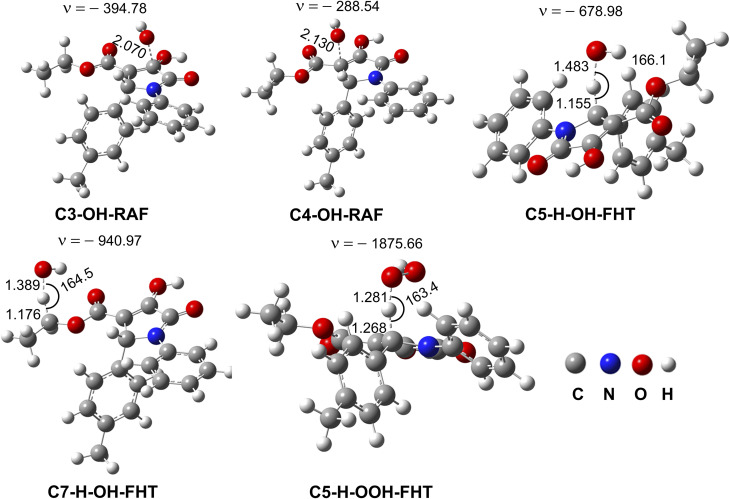
Optimized geometries of the main transition states between 4b and HO˙/HOO˙ radicals according to FHT and RAF processes.

The radical adduct formation (RAF) process also plays a key role in the radical scavenging of compounds with double bonds, particularly in the HO˙ antiradical action, as demonstrated by prior research^62,69–71^ and hence this pathway should be also examined. Gibbs free energy changes (Δ*G*°) for the HO˙ and HOO˙ radical scavenging reactions of 4b*via* the FHT, SET and RAF mechanisms were calculated in the gas phase and is presented in Table 5. With the exception of the SET reaction (Δ*G*° = 158.8 kcal mol^−1^), it was found that the HO˙ radical scavenging reactions are spontaneous (Δ*G*° < 0) for all positions in 4b; however, the HOO˙ radical scavenging is spontaneous only at the C5–H bond (Δ*G*° = −10.2 kcal mol^−1^) according to the FHT mechanism. Consistently all positions (Δ*G*° < 0, Table 5) were evaluated kinetically for the radical scavenging of 4b against the HO˙ radical in a vacuum, while only the H-abstraction of the C5–H bond was estimated for the HOO˙ radical. The results are presented in Table 6 and Fig. 2.

**Table 5:** The calculated Δ*G*° values (in kcal mol^−1^) of the reactions of 4b with HO˙ and HOO˙ following the FHT, SET and RAF mechanisms

Mechanisms	Positions	Δ*G*°	
		HO˙	HOO˙
FHT	O3–H	−22.8	8.5
	C5–H	−41.5	−10.2
	C7–H	−21.0	10.2
	C8–H	−15.8	15.4
	C21–H	−25.8	5.4
RAF	C3	−24.8	6.3
	C4	−25.0	3.2
	C9	−6.7	
	C10	−11.5	
	C11	−4.5	
	C12	−7.4	
	C13	−4.4	
	C14	−8.4	
	C15	−8.6	
	C16	−6.2	
	C17	−6.3	
	C18	−9.3	
	C19	−7.1	
	C20	−5.1	
SET		158.8	163.5

**Table 6:** Calculated activation energies (Δ*G*^≠^ (kcal mol^−1^)), tunneling corrections (*κ*) and *k*_Eck_, *k*_overall_ (M^−1^ s^−1^) and branching ratios (*Γ*, %) at 298.15 K for the HO˙ and HOO˙ scavenging of 4b

Mechanisms	Positions	HO˙				HOO˙		
		Δ*G*^≠^	*κ*	*k*_Eck_	*Γ*	Δ*G*^≠^	*κ*	*k*_Eck_
FHT	O3–H	10.7	21.3	1.93 × 10^6^	0.0			
	C5–H	3.4	1.1	2.19 × 10^10^	37.9	18.0	43.8	5.48 × 10^1^
	C7–H	5.0	1.0	1.39 × 10^9^	2.4			
	C8–H	9.4	2.7	2.11 × 10^6^	0.0			
	C21–H	6.8	1.1	7.23 × 10^7^	0.1			
RAF	C3	4.8	1.0	1.93 × 10^9^	3.3			
	C4	3.2	1.1	3.18 × 10^10^	55.1			
	C9	10.2	1.3	2.89 × 10^5^	0.0			
	C10	5.5	1.1	6.43 × 10^8^	1.1			
	C11	9.2	1.3	1.51 × 10^6^	0.0			
	C12	7.9	1.2	1.26 × 10^7^	0.0			
	C13	9.8	1.3	5.36 × 10^5^	0.0			
	C14	7.9	1.1	1.20 × 10^7^	0.0			
	C15	9.7	1.1	5.06 × 10^5^	0.0			
	C16	7.4	1.1	5.06 × 10^5^	0.0			
	C17	7.6	1.2	5.06 × 10^5^	0.0			
	C18	8.0	1.0	5.06 × 10^5^	0.0			
	C19	6.8	1.1	5.06 × 10^5^	0.0			
	C20	7.4	1.2	5.06 × 10^5^	0.0			
*k*_overall_				**5.77 × 10** ^**10**^				**5.48 × 10** ^**1**^

The overall rate constant (*k*_overall_) for HO˙ radical scavenging in the gas phase was 5.77 × 10^10^ M^−1^ s^−1^, however only 5.48 × 10^1^ M^−1^ s^−1^ for HOO˙ antiradical activity (Table 6). The hydroxyl antiradical activity was defined by a combination of the RAF mechanism (at the C3 (*Γ* = 3.3%) and C4 (*Γ* = 55.1%)) and the FHT mechanism (at the C5–H (*Γ* = 37.9%) and C7–H (*Γ* = 2.4%)). The H-abstraction of the C5–H bond determined the HOO˙ antiradical activity while contributing 37.9% to the total HO˙ radical scavenging. Thus these reactions should be used for further kinetic evaluation in the physiological environments.

##### 3.2.2.2. The antiradical activity in the physiological environments

###### 3.2.2.2.1. Acid base equilibrium

**Fig. 3:**
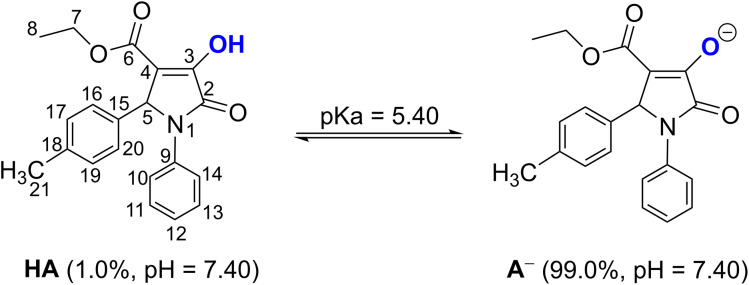
The deprotonation of 4b at pH = 7.40.

In order to account for the influence of physiological settings, the radical scavenging of 4b against HO˙ and HOO˙ radicals was simulated in water at pH = 7.4 for the aqueous solution and in pentyl ethanoate for lipid medium.^40,55^ Following the literature,^72,73^ the acid-base equilibria of 4b at the O3–H bond were computed to estimate the state of 4b in aqueous solution at pH = 7.4 (Fig. 3). The p*K*_a_ value was 5.40. Fig. 3 shows that 4b consistently exists both in the neutral state (HA, 1.0%) and the monoanion state (A^−^, 99.0%) at physiological pH (7.40). These states are employed for further investigation in the polar medium.

The QM-ORSA protocol was used to evaluate the kinetics of the HO˙ and HOO˙ scavenging reactions in physiological environments.^40^ The results are shown in Table 7. The results showed that in pentyl ethanoate and water solvents, the *k*_overall_ values for the 4b + HO˙ + reaction were 2.05 × 10^9^ and 1.54 × 10^10^ M^−1^ s^−1^, respectively, while those were 6.90 × 10^−1^ and 1.82 × 10^3^ M^−1^ s^−1^ for the 4b + HOO˙ reaction. The HO˙ antiradical activity in lipid medium was defined by the RAF mechanism at the C4 position (*Γ* = 57.1%) and the FHT pathway at C5–H bond (*Γ* = 30.6%), whereas that for the aqueous solution was characterized by the SET reaction of the anion state (*Γ* = 56.7%). The RAF and FHT reactions of the anion state contributed more than 40% in the hydroxyl antiradical activity in water at pH = 7.40, however these reactions of the neutral state had no contribution to the activity. At the same time, the FHT (C5–H, *Γ* = 59.7%) and RAF (C4, *Γ* = 40.2%) reactions of the anion state played a decisive role in the HOO˙ antiradical activity of 4b in water at pH = 7.40. The ability of 4b to scavenge hydroxyl radical in polar and non-polar environments is comparable to that of common antioxidants such melatonin,^74^ gallic acid,^75^ indole-3-carbinol,^71^ ramalin,^70^ and Trolox.^40^ However, 4b exhibited low hydroperoxyl radical scavenging activity in both the lipid and polar media. Thus 4b is a good but not exceptional hydroxyl radical scavenger in the physiological environment with targeted activities that may invite further studies into its activity against specific compounds.

**Table 7:** Gibbs free energies of activation (Δ*G*^≠^, kcal mol^−1^), rate constants (*k*_app_, *k*_f_, M^−1^ s^−1^) and branching ratios (*Γ*, %) at 298.15 K, in the NPY oxidation by HO˙/HOO˙ radicals in the studied environments

States		Mechanisms		Pentyl ethanoate			Water				
				Δ*G*^≠^	*k*_app_	*Γ*	Δ*G*^≠^	*k*_app_	*f*	*k*_f_	*Γ*
HO˙	HA	SET					22.8	1.20 × 10^−4^	0.01	1.20 × 10^−6^	0.0
		FHT	C5–H	5.5	6.27 × 10^8^	30.6	5.6	6.76 × 10^8^	0.01	6.76 × 10^6^	0.0
			C7–H	6.6	1.19 × 10^8^	5.8	8.2	8.10 × 10^6^	0.01	8.10 × 10^4^	0.0
		RAF	C3	6.5	1.32 × 10^8^	6.4	6.3	1.95 × 10^8^	0.01	1.95 × 10^6^	0.0
			C4	5.2	1.17 × 10^9^	57.1	4.7	3.08 × 10^9^	0.01	3.08 × 10^7^	0.2
	A^−^	SET					0.7	8.80 × 10^9^	0.99	8.71 × 10^9^	56.7
		FHT	C5–H				3.8	2.38 × 10^9^	0.99	2.35 × 10^9^	15.3
			C7–H				6.1	2.13 × 10^8^	0.99	2.11 × 10^8^	1.4
		RAF	C3				∼0	2.10 × 10^9^	0.99	2.08 × 10^9^	13.5
			C4				∼0	2.00 × 10^9^	0.99	1.98 × 10^9^	12.9
	*k*_overall_				**2.05 × 10** ^**9**^					**1.54** × **10**^**10**^	
HOO˙	HA	FHT	C5–H	20.6	6.90 × 10^−1^	100.0	19.9	3.10	0.01	3.10 × 10^−2^	0.0
	A^−^	SET					19.3	4.80 × 10^−2^	0.99	4.75 × 10^−2^	0.0
		FHT	C5–H				15.0	1.10 × 10^3^	0.99	1.09 × 10^3^	59.7
		RAF	C3				17.5	1.10	0.99	1.09	0.1
			C4				13.6	7.40 × 10^2^	0.99	7.33 × 10^2^	40.2
	*k*_overall_				**6.90 × 10** ^**−1**^					**1.82** × **10**^**3**^	

## 4. Conclusion

Six polysubstituted 3-hydroxy-3-pyrroline-2-ones were successfully synthesized by using three-component reactions at room temperature. The DPPH assay indicated that 4b exhibits the highest DPPH radical scavenging activity. The thermodynamic and kinetic calculations also showed that 4b is a potent HO˙ radical scavenger with *k*_overall_ = 2.05 × 10^9^ and 1.54 × 10^10^ M^−1^ s^−1^ in pentyl ethanoate and water, respectively. However, 4b exhibited only low hydroperoxyl radical scavenging activity in either of the lipid and polar media. Compared to typical antioxidants such as melatonin, gallic acid, indole-3-carbinol, ramalin, and Trolox, the ability of 4b to scavenge hydroxyl radicals in polar and non-polar environments is similar to that of these compounds. Thus 4b is a promising HO˙ radical scavenger in physiological environments.

## Conflicts of interest

There are no conflicts to declare.

## Supplementary Material
